# All things are active … new approach methods for excipient safety

**DOI:** 10.3389/fphar.2026.1896544

**Published:** 2026-09-09

**Authors:** Thomas Hartung

**Affiliations:** 1 Center for Alternatives to Animal Testing (CAAT), Johns Hopkins Bloomberg School of Public Health, Baltimore, MD, United States; 2 CAAT-Europe, University of Konstanz, Konstanz, Germany

**Keywords:** context of use, excipients, exposure science, immunotoxicity, inactive ingredients, nanomedicine, New Approach Methodologies (NAMs), pediatric safety

## Abstract

Excipients make up the majority of most medicines by mass, yet they are still called “inactive,” still largely qualified against a 2005 guidance, and still assessed product by product with animal studies that answer the wrong questions. The past decade has dismantled the “inactive” assumption: polyethylene glycol and polysorbate anaphylaxis, benzyl alcohol gasping syndrome, propylene glycol acidosis, benzalkonium bronchospasm, and the recurrent diethylene glycol mass poisonings of children all show that excipient risk is real, but that it is contextual rather than intrinsic. I argue that this contextual character makes excipients an almost ideal proving ground for New Approach Methodologies (NAMs). Excipient hazard depends on route, dose, duration, formulation, impurities, and patient population—exactly the variables that exposure science, computational toxicology, and human *in vitro* systems handle well and that whole-animal testing handles poorly. I propose replacing the test-list mentality with a context-of-use excipient safety passport, sketch where NAMs already carry decisions and where they only inform them and offer a phased implementation agenda. The goal is to convert excipient safety from a static checklist into a human-relevant, exposure-led, mechanistic discipline.

## 1 Introduction

Open almost any medicine and weigh what is inside. In the great majority of tablets, capsules, syrups, and injectables, the active pharmaceutical ingredient is a minority component, sometimes down to a rounding error. The rest—by some industry estimates, 80%–90% of the product by mass for many solid oral dosage forms, though this fraction varies widely across routes, biologics, and advanced delivery systems—is excipient: fillers, binders, disintegrants, solvents, surfactants, preservatives, buffers, colorants, coating polymers, and increasingly the lipids and polymers that build nanoscale delivery systems (Sheskey et al., 2020; Giffen et al., 2023). We call these materials “inactive.” The word is a convenience that has hardened into a doctrine; for a growing set of materials, the doctrine is wrong. The point is not that most excipients are hazardous, but that the blanket assumption of inactivity is unsafe: certain excipients have well-established biological activity, and how frequently clinically consequential excipient toxicity occurs across products is itself an open, under-quantified question. Paracelsus (1493–1541) already taught 500 years ago, “All things are poison, and nothing is without poison; only the dose makes a thing not a poison.” We will see how much he ‘nailed it’ in this case as well.

The global excipients market is projected to grow from approximately USD 11.0 billion in 2025 to USD 14.9 billion by 2030: a compound annual growth rate near 6%, driven by patient-centric formulations, generics, and the search for functional novel excipients (MarketsandMarkets, 2025). Behind that number sits a quiet expansion of what excipients are asked to do. They now solubilize the insoluble, mask taste, extend release, stabilize biologics, and ferry messenger RNA past the cell membrane. The materials enabling these feats are not the inert sugars and starches of the pharmacopoeial imagination. They are surfactants, ionizable lipids, methacrylate copolymers, PEGylated species, and engineered nanoparticles whose biological behavior depends on size, charge, and the company they keep. The category is also heterogeneous in origin: biologically derived excipients such as gelatin, lanolin, and cochineal-derived carmine bring source variability, sensitization potential, and compositional complexity that inert sugars and starches do not.

The assumption of inactiveness has been falsified repeatedly, and sometimes catastrophically. Polyethylene glycol (PEG), long treated as the very definition of an inert spacer, became a prime suspect in anaphylaxis to messenger RNA COVID-19 vaccines and is now recognized as a hidden cause of reactions to multiple unrelated drugs (Stone et al., 2019; Malkawi and Altahrawi, 2025). Benzyl alcohol, a humble preservative, killed premature neonates through “gasping syndrome” because immature livers could not metabolize it (Clouser and Diseroad, 2022). Propylene glycol, a workhorse solvent, produces lactic acidosis and renal injury when infused in quantity to patients who cannot clear it (Clouser and Diseroad, 2022). In the most damning failures of all, glycerin and propylene glycol contaminated with diethylene glycol have poisoned children in The Gambia, Indonesia, Uzbekistan, and elsewhere, with more than 300 deaths reported in 2022 alone (WHO, 2022; WHO, 2023). The 1937 sulfanilamide disaster, which gave the United States its modern drug law, was a diethylene glycol poisoning. The same molecule in the same class of excipient was still killing children 85 years later. That is not a knowledge problem—it is a systems problem.

The regulatory scaffolding for excipient safety has not kept pace. The US Food and Drug Administration’s nonclinical excipient guidance dates from 2005, and there is still no internationally harmonized framework for qualifying a genuinely novel excipient (FDA, 2005; Giffen et al., 2023). When the International Consortium for Innovation and Quality in Pharmaceutical Development surveyed 33 novel-excipient programs, only two reported using New Approach Methodologies (NAMs) at all, and both used them alongside, not instead of, *in vivo* studies (Giffen et al., 2023). Uptake is, in other words, near zero, at precisely the moment when the science is ready and the need is acute.

My thesis here is that excipients are not a difficult case for NAMs but an unusually favorable one. The reason is conceptual. For most excipients, safety is not an intrinsic property of the molecule, a fixed “safe” or “unsafe” stamp. It is, rather, a function of context: the route of administration, the concentration at the site of contact, the maximum daily exposure summed across every product a patient takes, the duration of treatment, the formulation it sits in, the impurities it carries, and the vulnerability of the patient receiving it. These are exactly the variables that exposure modeling, computational chemistry, and human-relevant *in vitro* systems were built to handle, and exactly the variables that a 90-day rat study handles poorly. For excipient endpoints specifically, the limited predictability of animal models and the closer human relevance of *in vitro* systems are documented in Szebeni (2014) and Kozma et al. (2019). This is the argument I have made for toxicology in general: mechanism and exposure should replace the animal as the unit of inference (Hartung and Leist, 2008; Hartung, 2009). Excipients are where that argument is easiest to win.

This is also a One Health and exposome question. A patient does not receive an excipient in isolation: they receive the same polysorbate, propylene glycol, or PEG across several medicines at once, and a neonate in intensive care may accumulate dozens of excipient exposures from necessary treatments (Nellis et al., 2015). The unit that matters is the cumulative human exposure, not the single dossier. I have argued elsewhere that integrating exposure into a human exposome framework is the missing half of modern toxicology, and excipients are a concrete, tractable place to demonstrate it (Hartung, 2023).

**FIGURE 1:**
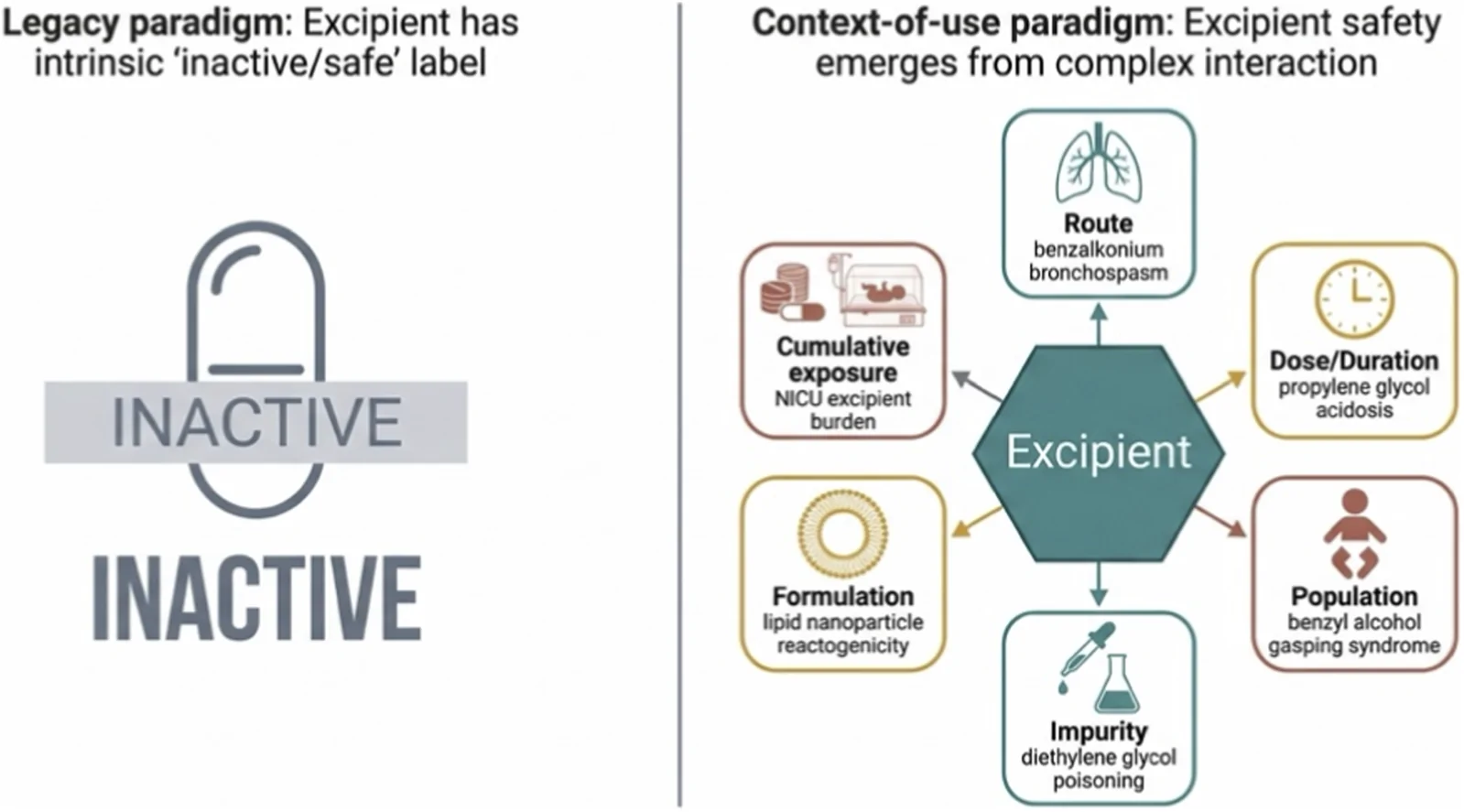
The inactive-ingredient fallacy: from intrinsic label to context of use. The figure contrasts the legacy paradigm, in which an excipient carries a fixed “inactive/safe” stamp, with the proposed context-of-use paradigm, in which safety emerges from the interaction of the material with route, dose, duration, formulation, impurity profile, and patient population. The left panel shows a single excipient molecule labeled “inactive”; the right panel shows the same molecule at the center of six radiating determinants, each annotated with a real failure example (route: benzalkonium bronchospasm; dose/duration: propylene glycol acidosis; population: benzyl alcohol gasping syndrome; impurity: diethylene glycol poisoning; formulation: lipid nanoparticle reactogenicity; cumulative exposure: NICU excipient burden). A further determinant, the material’s own physicochemical attributes (particle size, surface charge, molecular-weight distribution, and reactivity), is shown interacting with these contextual variables so that the diagram does not imply that risk arises from context alone; dose/duration denotes the per-product regimen, while cumulative exposure denotes the summed exposure across all products, and the figure and legend use these terms consistently with the text. The visual thesis is that the interaction of material properties with the contextual variables is the natural domain of NAMs.

**FIGURE 2:**
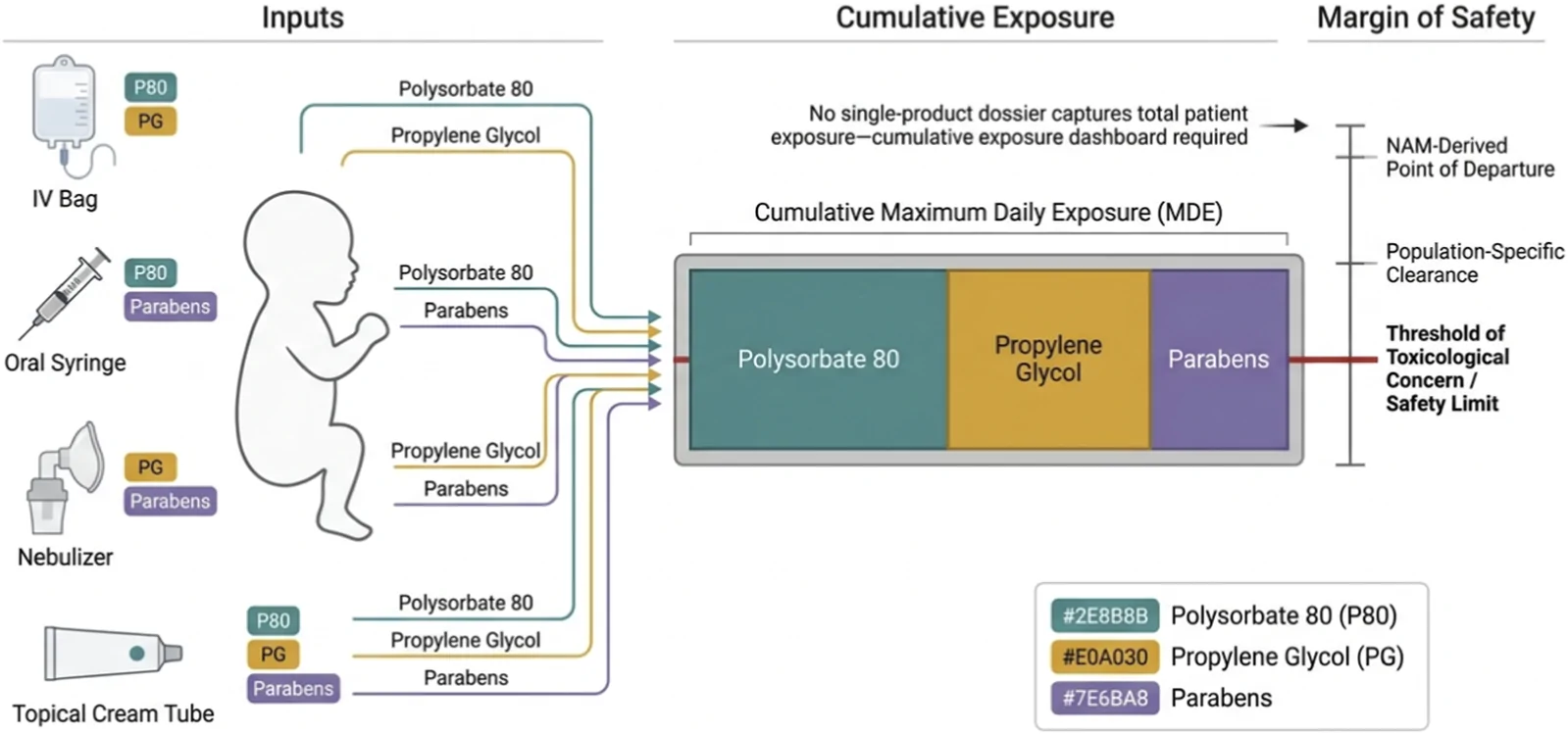
Cumulative excipient exposure: why the unit of concern is the patient, not the product. A schematic of a single patient (a NICU neonate is the worked example) receiving multiple products simultaneously, with the same excipient (polysorbate 80, propylene glycol, and parabens) arriving through several routes. Cumulative exposure is computed separately for each excipient, because these substances have different units, kinetics, and points of departure and cannot be summed into a single toxicological quantity; where combined effects are of interest, a component-based approach such as a hazard index is used. Each excipient is compared against its own NAM-derived point of departure and margin, and population-specific clearance is shown as a modifier that is most informative for systemically acting excipients, with the relevant exposure metric depending on route and endpoint for local effects. The figure shows that no single product dossier observes the total, which is the gap a cumulative exposure dashboard closes.

What follows is a deliberately practical map. Figures 1, 2 summarize the two organizing concepts developed below: context-of-use safety and cumulative patient-level excipient exposure. I first explain why the context-of-use logic of excipients fits NAMs so well and why we should begin with a structured safety passport rather than a test list (Section 2). I then summarize the decade of evidence that excipients cause real harm, because the case for change rests on it (Section 3). The core of the article examines where NAMs add the most value: exposure science (Section 4), impurities and degradants (Section 5), and the route-specific modules for local tolerance, parenteral immunotoxicity, inhalation, and nanocarriers (Section 6). I then treat vulnerable populations (Section 7) and systemic organ toxicity (Section 8), assemble the pieces into an integrated package and weight-of-evidence framework (Section 9), argue for excipient-aware pharmacovigilance (Section 10), and close with an implementation agenda and a candid account of the risks of getting this wrong (Section 11).

## 2 Why excipients are a special NAM opportunity

The intellectual error embedded in the word “inactive” is the assumption that an excipient has a safety status the way it has a molecular weight. It does not. The single most useful concept in excipient safety is context of use, and this is the concept on which the entire modern NAM enterprise turns. Context of use does not displace the material; it acts on it. Particle size, surface charge, chemical reactivity, surfactant behavior, persistence, molecular-weight distribution, and immunogenic motifs are intrinsic, material-dependent properties, so excipient risk is most accurately written as material properties times the context of use rather than as intrinsic safety versus contextual safety.

Consider how the existing system already half-admits this. The FDA’s Inactive Ingredient Database (IID^1^) lets developers reference prior use of an excipient at a given route and level, which is genuinely useful when a new product stays inside that envelope. But the FDA’s own guidance cautions that the listed “maximum potency” is not a maximum daily exposure and certainly not a permitted daily intake (FDA, 2019). The moment a sponsor wants a higher level, a different route, or a longer duration, the database entry stops answering the question. What is needed at that boundary is not another animal study but a quantitative argument about exposure and mechanism. That is a NAM argument.

The 2005 FDA nonclinical excipient guidance, still the reference text, was written for a world of small-molecule fillers and preservatives (FDA, 2005). It recognized, to its credit, that prior human exposure, food or GRAS experience, route, duration, and patient population can justify very different nonclinical packages. That insight is correct and underused. However, the guidance predates microphysiological systems, the maturation of physiologically based pharmacokinetic (PBPK) modeling, the ICH M7 framework for mutagenic impurities, and lipid nanoparticles as a mainstream modality. The conceptual seed of context of use is in the 2005 text; the tools to operationalize it arrived afterward.

The timing now is unusually good because the regulatory posture is shifting. The FDA’s 2025 roadmap to reduce animal testing^2^ and its draft framework on the use of NAMs^3^ articulate four elements for the regulatory acceptance of a NAM: a clearly defined context of use, human biological relevance, technical characterization, and fit-for-purpose performance (FDA, 2025; Hartung, 2025; Hansell and Hartung, 2025a; Hansell and Hartung, 2025b). Crucially, the draft states that a NAM need not be fully validated in the classical sense to be used: a fit-for-purpose method can address a specific toxicological concern within a weight-of-evidence package. Fit-for-purpose is not a license to skip evidence of reliability; a method still needs demonstrated transferability and reproducibility and, where feasible, concordant results across laboratories, so that a NAM-based conclusion does not rest on a single unbenchmarked assay (Kopańska and Hartung, 2026). For excipients, where the questions are narrow and mechanistic (“does this surfactant activate complement in human serum?”, “does this residual monomer carry a structural alert for mutagenicity?”), fit-for-purpose is exactly the right standard. This description fits many excipient questions well; it fits less comfortably for chronic exposure, developmental effects, complex mixtures, poorly characterized polymers, and multifunctional nanocarriers, where broader biological questions remain and the fit-for-purpose context must be defined case by case. Table 1 shows that NAMs correspond to the different problems recorded in recent years.

**TABLE 1:** Documented excipient safety signals and the most relevant method or risk-control approach for each.

Excipient	Failure mode	Population/route	Most relevant method/risk-control approach
Polyethylene glycol (PEG)	Immediate hypersensitivity/anaphylaxis	Vaccines, multiple drugs; IV/IM, oral	Complement activation, basophil/mast-cell activation, whole-blood cytokine release
Polysorbate 80	Hypersensitivity; cross-reacts with PEG	IV biologics, chemotherapy	Complement activation, basophil/mast-cell activation, whole-blood cytokine release
Cremophor EL	Infusion anaphylaxis (∼2% severe)	IV paclitaxel; oncology	Immune panel; reformulation (albumin-bound)
Benzyl alcohol	Gasping syndrome (metabolic acidosis)	Neonates; IV, flush solutions	Age-specific PBPK; cumulative exposure model
Propylene glycol	Lactic acidosis, renal injury, CNS depression	ICU infusions, neonates, impaired clearance	PBPK/IVIVE; exposure modeling
Ethanol	Acute CNS and metabolic toxicity	Young children; oral, IV co-solvent	Age-specific PBPK
Benzalkonium chloride	Bronchospasm; corneal toxicity	Asthmatics, chronic ocular use; inhaled, ocular	ALI airway cultures; RhCE ocular models
Sulfobutylether-β-cyclodextrin	Renal accumulation	Renal impairment; IV	Proximal-tubule models; PBPK
Titanium dioxide	Nanoparticle genotoxicity concern	Oral; broad use	*In vitro* genotoxicity battery with dispersion, dosimetry, uptake and agglomeration controls; particle physicochemical characterization; assay-interference checks
DEG-contaminated glycerin/PG	Acute kidney injury, death	Children; oral syrups	Batch identity and purity testing (a quality, not a NAM, gap)

The table operationalizes the article’s claim that most of these failure modes are mechanistically interpretable while recognizing that retrospective interpretability is not the same as prospective predictability. The final column lists methods or, where relevant, quality and risk-management measures rather than NAMs alone.

There is a structural reason for why excipients have been left behind, and naming it points to the solution. Much excipient safety information resides in supplier files and Drug Master Files, fragmented across companies, so that sponsors routinely repeat studies they could have inherited. An IQ Consortium survey found not only near-zero NAM uptake but recurring pain around defining impurities in heterogeneous materials and bridging changes in synthetic route, site, or specification (Giffen et al., 2023). These are information-architecture problems as much as toxicology problems, and NAMs plus a shared data model solve both.

I therefore propose, as the organizing idea of this study, that a NAM-based excipient program should not begin with a list of assays to run. Instead, it should begin with a structured excipient safety passport: a living document that states the material’s identity and attributes, its context of use, its prior evidence, an explicit exposure model, the relevant hazard modules, and an integrated conclusion with a defined point of departure, margins of safety, proposed specifications, and a list of residual uncertainties that actually require new testing. The passport reframes the question from “Which tests are mandatory?” to “What do we need to know to make this decision, and what is the most human-relevant way to know it?” Everything else discussed here is content for that passport. Architecturally, I envisage a common core dataset shared by all excipients (identity and attributes, exposure, impurity control, and an integrated conclusion) plus class-specific modules that differ for chemically simple excipients, heterogeneous polymers, biologically derived materials, and nanocarriers. The passport must also state whether it describes the excipient as a generic chemical entity, a defined grade or specification, a manufacturer-specific material, or within a particular formulation, because manufacturing and composition can materially change the evidence base.

## 3 The harm is real: a decade of excipient safety signals

A call for new methods is only persuasive if the old assumption has demonstrably failed. It has. The past 10 years have produced a steady accumulation of evidence, across three categories, that excipients can harm patients. I summarize it here not for completeness but because each failure mode points to a specific NAM that could have anticipated it. These examples establish clinical plausibility and importance; they do not by themselves establish the overall prevalence or population burden of excipient-related harm, which remains poorly quantified. Where robust incidence estimates are lacking, I treat them as sentinel events rather than as measures of how commonly excipient toxicity occurs.

### 3.1 Hypersensitivity and pseudoallergy

Excipients are now recognized triggers of immediate hypersensitivity, both IgE-mediated and non-immune “pseudoallergic” reactions, presenting as urticaria, angioedema, bronchospasm, or anaphylaxis (Malkawi and Altahrawi, 2025). PEG is the emblematic case. Once the definition of inertness, it emerged during the COVID-19 vaccine rollout as a credible cause of rare anaphylaxis, prompting contraindications for known PEG allergy, and it is increasingly invoked to explain patients who react to several structurally unrelated drugs (Stone et al., 2019; Malkawi and Altahrawi, 2025). Polysorbate 80, chemically adjacent, can cross-react. Cremophor EL (polyoxyl 35 castor oil), the solvent in conventional paclitaxel, is not an inert vehicle but a documented cause of severe anaphylactoid infusion reactions, which, in historically reported series, occurred in roughly 40% of patients overall and as life-threatening anaphylaxis in approximately 2%, despite routine premedication (figures that predate current infusion and premedication practice), which is why albumin-bound paclitaxel, a reformulation that simply removes the offending excipient, was such an advance (Gelderblom et al., 2001; Picard and Castells, 2015). Parabens, azo dyes such as tartrazine, sulfites, gelatin, and carboxymethylcellulose round out a now well-documented list (Malkawi and Altahrawi, 2025). Some analyses suggest that a meaningful fraction of otherwise idiopathic anaphylaxis may be excipient-related and simply misattributed to the active drug (Malkawi and Altahrawi, 2025).

The mechanistic lesson is precise. These are human immune phenomena, and animal models predict them poorly. They are, however, increasingly tractable in human *in vitro* systems: complement activation in human serum, basophil and mast-cell activation tests and in whole-blood cytokine release assays. The reaction that surprised the world in 2021 was not, in principle, unpredictable.

### 3.2 Direct and organ-specific toxicity

The second category is dose-dependent toxicity, which becomes visible when excipients accumulate, when elimination is impaired, or when the patient is small. Benzyl alcohol causes neonatal gasping syndrome, a metabolic acidosis with cardiovascular collapse, because newborns cannot convert its metabolite benzoic acid to hippuric acid; the EMA contraindicates it under 3 years of age (Clouser and Diseroad, 2022). Propylene glycol, metabolized through alcohol dehydrogenase, produces an osmolar gap, lactic acidosis, CNS depression, and acute kidney injury when delivered in grams per day, classically through the continuous infusion of PG-solubilized sedatives in the intensive care unit (Clouser and Diseroad, 2022). Ethanol, still present in some pediatric and parenteral formulations, is poorly metabolized by young children. Benzalkonium chloride, a preservative in nebulizer solutions, nasal sprays, and eye drops, is a dose-dependent bronchoconstrictor in asthmatics and a corneal toxicant with chronic ocular use (Prabhakaran et al., 2017). Sulfobutylether-beta-cyclodextrin, an elegant solubilizer for intravenous drugs, is renally cleared and accumulates in renal impairment, which is why intravenous voriconazole carries a creatinine-clearance restriction (Luke et al., 2010).

Most of these are, at root, a quantitative exposure and target-tissue-response story: how much reaches the target tissue, in whom, and for how long? For systemically acting examples (benzyl alcohol, propylene glycol, ethanol, and renally cleared cyclodextrins), this is largely pharmacokinetic; for benzalkonium-associated bronchoconstriction and chronic corneal injury, local concentration, epithelial interaction, and formulation characteristics can matter at least as much as systemic pharmacokinetics. That is the natural domain of PBPK and *in vitro* to *in vivo* extrapolation (IVIVE), not of default uncertainty factors.

### 3.3 Impurities, degradants, and contamination

The third category is the deadliest and, paradoxically, the most preventable. The diethylene glycol poisonings are not the toxicity of an excipient as intended but a failure of excipient quality, and they belong squarely in any honest account of excipient safety. The 2022 to 2023 episodes in The Gambia, Indonesia, and Uzbekistan, with over 300 child deaths, were traced to industrial-grade glycerin and propylene glycol contaminated with diethylene and ethylene glycol (WHO, 2022; WHO, 2023). Less lethal but more pervasive are nitrosamines formed when nitrite impurities in excipients react with amine drug substances, ethylene oxide residues, peroxides, aldehydes, residual metals and solvents, and leachables from packaging. Titanium dioxide, a near-ubiquitous colorant, was judged no longer safe as a food additive in the EU over nanoparticle genotoxicity concerns, though a feasible wholesale replacement in medicines has not yet been found (EFSA Panel on Food Additives and Flavourings, 2021; Haigney, 2025).

These failures can, with one exception, be addressed by chemistry-based NAMs that already exist and are already accepted: structure-based mutagenicity prediction, nitrosamine risk assessment, and route-aware impurity limits. This maturity is real for defined small-molecule impurities with a known structure; it does not extend equally to nanoparticulate titanium dioxide, complex or unidentified degradants, leachables, peroxides and aldehydes, or incompletely characterized polymers and mixtures, which require broader analytical, physicochemical, and biological characterization before any computational prediction applies. The exception, contamination by the criminal or negligent substitution of industrial solvents, is not a toxicology gap at all; it is a supply-chain and analytical-testing gap, and no NAM replaces testing every batch for identity and purity.

The throughline across all three categories is that the harm was contextual and, in most cases, mechanistically interpretable in retrospect. Retrospective explainability is not the same as prospective predictability, and I do not claim that every one of these events could have been foreseen before clinical exposure; the honest claim is that a mechanistic, exposure-led framework would have raised the right questions earlier for many of them. That is the case for NAMs, made by the failures themselves.

## 4 Exposure science: the most immediate opportunity

If I had to choose the single highest-yield investment in excipient NAMs, it would not be a fashionable organ-on-chip. It would be exposure science, because for excipients, the missing number is almost always the exposure, not the hazard.

The reason is structural and specific to excipients. Unlike an active ingredient, which a patient generally receives from one product at a known dose, an excipient may arrive from many products at once. A patient on five medicines may be receiving the same polysorbate or propylene glycol five times, and no single dossier sees the total. The International Pharmaceutical Excipients Council’s safety guidance makes exactly this point: excipient exposure differs from both drugs and foods because the same material reaches the patient across multiple dosage forms and indications, with dose, duration, frequency, route, and population as the core determinants (IPEC, 2021). The unit of concern is cumulative, and cumulative exposure is, in principle, quantifiable when sufficiently detailed formulation and use data are available. In practice, quantification is constrained by incomplete disclosure of excipient amounts, manufacturer-specific formulation differences, lot variability, uncertain adherence, and concurrent exposure from several routes; these are not reasons to abandon an exposure-led framework but variables it must explicitly represent through conservative assumptions and stated uncertainty ranges where data are missing.

Four exposure tools deserve immediate, systematic deployment. The first is route-specific maximum daily exposure modeling. For oral, parenteral, inhaled, dermal, ocular, nasal, and vaginal routes, one can link IID entries, labels, regimens, and population body weights to derive realistic and worst-case daily exposures, replacing the false comfort of a single “maximum potency” figure. The second is PBPK and IVIVE for special populations. Neonates, the renally and hepatically impaired, pregnant patients, and the critically ill differ in body water, clearance, protein binding, and barrier maturity in ways that PBPK represents transparently (Tsaioun et al., 2016) and that default animal-derived uncertainty factors represent crudely (Hartung, 2018). The third is local rather than only systemic exposure. For inhaled, nasal, dermal, ocular, and gastrointestinal excipients, toxicity is often driven by local concentration, osmolality, surfactant activity, pH, residence time, or epithelial barrier disruption, none of which a plasma concentration captures. The fourth is cumulative excipient exposure dashboards, which matter most in the settings where the burden is highest.

The neonatal intensive care unit is the proof case and the moral one. A pan-European point-prevalence study that actually counted found that hospitalized neonates routinely receive multiple potentially harmful excipients, including parabens, polysorbate 80, propylene glycol, benzoates, saccharin, sorbitol, ethanol, and benzalkonium chloride, and that marked regional differences in their use imply that excipient-free alternatives often exist and could be substituted (Nellis et al., 2015). These infants were not victims of a single overdose but of an unmonitored cumulative cocktail, and a cumulative exposure model linked to the formulary would have made the avoidable fraction visible.

The practical deliverable is modest and powerful: this is an excipient exposure qualification report that states, in plain terms, that at the proposed maximum daily exposure and route, the margin relative to prior human exposure and a NAM-derived point of departure is X, and that for the most vulnerable subgroup, the lowest clearance or highest mg/kg exposure gives margin Y. That single page reframes the entire qualification conversation around exposure—where it belongs.

## 5 Impurities, degradants, and manufacturing changes

The most mature NAM opportunity in all of excipient safety is the chemical risk assessment of impurities, where validated, regulator-accepted computational methods already carry decisions today.

The anchor is ICH M7, which governs DNA-reactive mutagenic impurities and accepts a two-model *in silico* strategy—one expert rule-based system and one statistical model—such that the absence of structural alerts from both can support a conclusion of no mutagenic concern without any new testing; positive or equivocal alerts trigger targeted Ames testing or a control strategy (ICH, 2023). This is a NAM that has been quietly making regulatory decisions for years, and it applies directly to the residual monomers, reagents, catalysts, degradants, and leachables that heterogeneous excipients carry. Many excipients are not single small molecules but polymers, surfactants, mixtures, fermentation products, or natural materials, and the IQ Consortium survey specifically flagged the difficulty of defining and quantifying their impurities (Giffen et al., 2023). Structure-based triage is precisely the tool for that heterogeneity. One caveat is essential: QSAR and structure-based triage require a known or reasonably defined structure. They cannot resolve unidentified chromatographic peaks or poorly characterized polymer fractions, so analytical identification and compositional characterization are prerequisites for, not secondary steps after, computational impurity assessment of heterogeneous excipients.

Three applications are immediately actionable. Mutagenic impurity triage applies ICH M7 QSAR to every residual species and confirms with Ames tests only when an alert fires. Nitrosamine risk assessment, now a major regulatory preoccupation, combines nitrite analytics in the excipient, reaction modeling, structure-based nitrosamine prediction, scavenger evaluation, and accelerated stability into a safe-by-design workflow; the FDA’s nitrosamine guidance explicitly identifies nitrite in excipients as a formation route (FDA, 2024). Elemental impurities and residual solvents are governed by ICH Q3D and Q3C, which already provide route-aware permitted daily exposures and a risk-assessment framework that includes excipient contributions (ICH, 2021; ICH, 2022).

The strategic payoff is in manufacturing comparability. When an excipient supplier changes process, site, catalyst, or purification, a common reflex, reported in industry experience and consistent with the IQ Consortium survey, has been to repeat *in vivo* studies (Giffen et al., 2023). A NAM package of analytical fingerprinting, impurity QSAR, targeted *in vitro* assays, and read-across can replace that reflex in many cases, with a stronger mechanistic basis than a repeat 28-day study. This is read-across logic (Ball et al., 2016), which I and others have argued is among the most consequential and underused tools in modern toxicology, applied to the impurity profile rather than the parent (Hartung, 2016; Luechtefeld et al., 2018; Rovida et al., 2020).

## 6 Route-specific NAM modules

**FIGURE 3:**
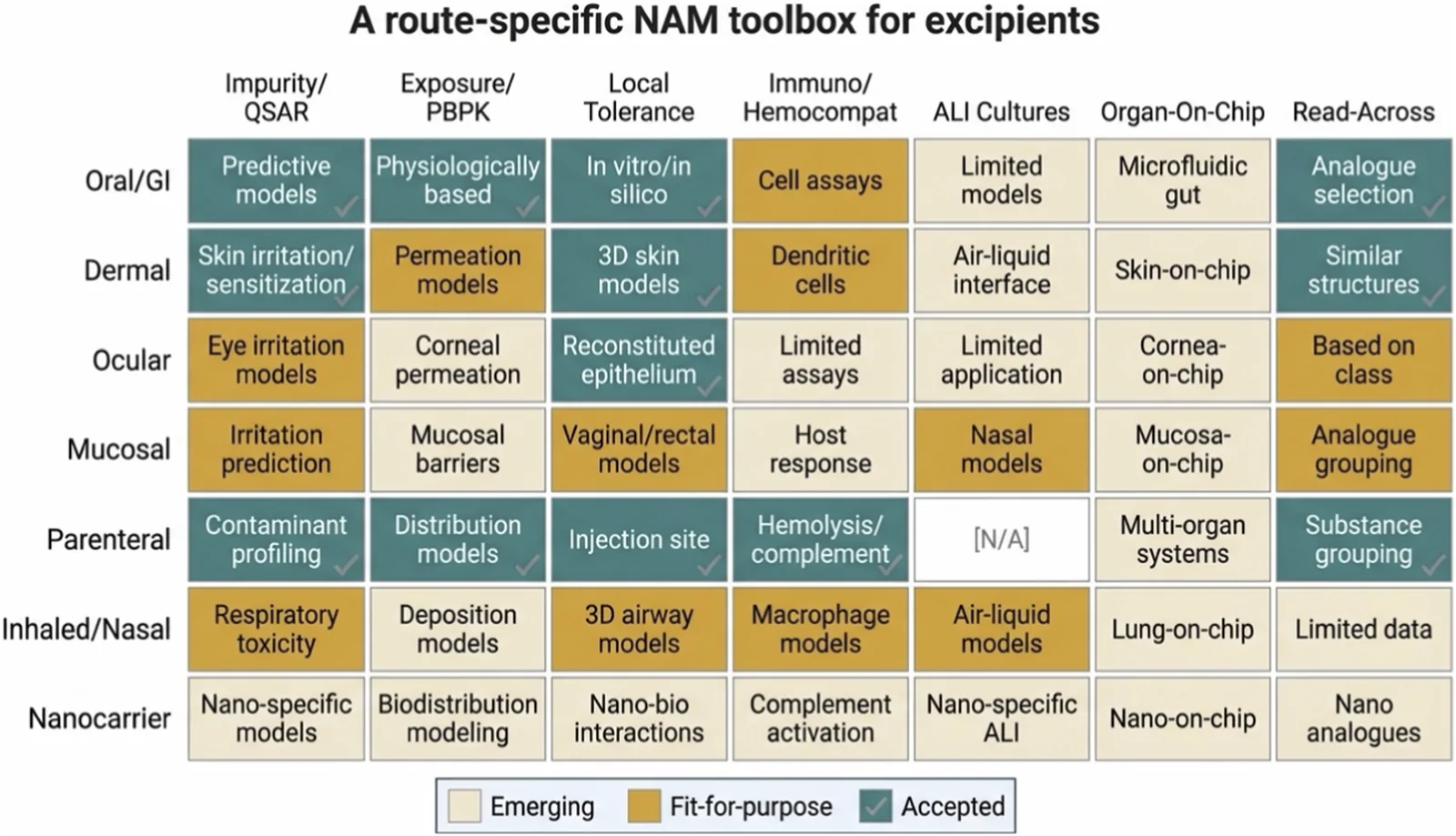
Route-specific NAM toolbox for excipients. Each cell names an item at a consistent level (a method, biological endpoint, model system, or evidence class) and is coded by scientific maturity and demonstrated applicability rather than by regulatory readiness as such, using three defined categories: accepted (OECD- or pharmacopoeia-recognized, for example, reconstructed-tissue local tolerance and the monocyte activation test for pyrogenicity), fit-for-purpose (usable within a weight-of-evidence package but requiring product-specific qualification), and emerging (promising but not yet standardized, as for much of the inhalation and nanocarrier work). Categories are conveyed by symbol as well as color, so the matrix remains interpretable in grayscale and for readers with color-vision deficiencies, and pyrogenicity/MAT is shown explicitly within the parenteral immuno/hemocompatibility module. The figure is the article’s strategic map of where to adopt versus where to qualify.

Beyond exposure and impurities, the substantive hazard work is route-specific, and the maturity of NAMs differs sharply by route (Figure 3). I treat the routes in descending order of NAM readiness.

### 6.1 Local tolerance: the routine cases

Dermal, ocular, and mucosal products are where excipient NAMs are closest to routine because the relevant OECD test guidelines are already accepted. Reconstructed human epidermis for skin irritation, *in chemico* and *in vitro* assays for skin sensitization within defined approaches, phototoxicity assays, and reconstructed human cornea-like epithelium for eye hazard (OECD TG 492B) are available now, and the FDA’s NAM framework explicitly recognizes that OECD guidelines are accepted for these endpoints (OECD, 2024; FDA, 2026). Penetration enhancers, solvents, surfactants, preservatives, adhesives, and viscosity agents can be screened in these systems, and for buccal, sublingual, vaginal, rectal, and nasal routes in human epithelial models with barrier-integrity and inflammatory readouts. Here the opportunity is not only to replace animal irritation tests but also to improve formulation, comparing candidate excipients, concentrations, and pH or osmolality windows before clinical irritation becomes a development problem. A caution on applicability domain belongs here: several of these methods were developed and validated for hazard classification of single substances, and an accepted irritation assay is not automatically valid for every concentration, formulation matrix, exposure duration, or specialized mucosal tissue. Using them for formulation-specific safety assessment requires confirming that the intended use falls within, or is bridged to, the method’s applicability domain.

### 6.2 Parenteral excipients: immunotoxicity and blood compatibility

Injectable excipients deserve a dedicated NAM strategy because their most important risks are human immune and blood-contact phenomena that animals predict poorly: complement activation, cytokine release, mast-cell and basophil activation, platelet activation, hemolysis, coagulation effects, and pyrogenicity. A near-term and already-realized win is pyrogenicity (Hartung, 2026). The European Pharmacopoeia has removed the rabbit pyrogen test from its monographs, with developers expected to select suitable *in vitro* methods such as the monocyte activation test (MAT) on a risk basis, through a new general chapter on pyrogenicity with implementation from 1 July 2025 (EDQM, 2024). This is a NAM displacing an animal test wholesale, by pharmacopoeial decision. I have argued for the MAT and for human *in vitro* pyrogenicity testing for three decades, and its arrival as the default is a model for how excipient NAMs should propagate (Hartung, 2015; Hartung, 2021; Hartung, 2026).

A strong parenteral panel pairs the MAT with human whole-blood cytokine release, complement activation in human serum or plasma, basophil and mast-cell activation, and hemocompatibility assays for hemolysis, platelet activation, and coagulation, with organ-specific co-culture follow-up where warranted; this is the *in vitro* immunotoxicology agenda I have argued is both necessary and feasible (Hartung and Corsini, 2013). This panel is especially relevant for PEGs, polysorbates, cyclodextrins, solubilizers, and lipid excipients, and it speaks directly to the COVID-19 vaccine experience, where PEG and polysorbate were central suspects, while skin testing proved to have poor predictive value and should not be treated as a definitive screen (Stone et al., 2019; Malkawi and Altahrawi, 2025). These assays differ in standardization: the monocyte activation test has an established pharmacopoeial performance standard, whereas complement activation, cytokine release, mast-cell responses, and assays for complex nanomaterials generally require product-specific qualification. The panel should state, for each excipient, which assays are used as validated methods and which as fit-for-purpose tools pending qualification.

### 6.3 Inhalation and nasal excipients

Inhaled excipients are an underdeveloped, high-impact opportunity precisely because animal respiratory anatomy and particle deposition diverge so strongly from the human lung. Carriers, surfactants, propellants, preservatives, absorption enhancers, and mucoadhesive polymers can cause local irritation, bronchoconstriction, barrier disruption, macrophage activation, or impaired mucociliary clearance. Human airway epithelial cultures at the air–liquid interface, nasal and bronchial organoids, alveolar epithelial and macrophage co-cultures, ciliary-beat and mucociliary-clearance assays, surfactant-function assays, and airway PBPK with deposition and dissolution modeling form a coherent human-relevant battery when it is organized around defined biological questions—deposition, epithelial injury, macrophage response, mucociliary clearance, dissolution, and persistence—rather than assembled as a catalogue of available technologies. The IQ survey is instructive: respiratory novel-excipient programs were among those that drew the most divergent and demanding regulatory feedback across regions (Giffen et al., 2023). That divergence is itself an argument for a shared, human-relevant inhalation NAM package.

### 6.4 Nanocarriers and complex delivery systems

Lipid nanoparticles, liposomes, polymeric nanoparticles, and PEGylated systems break the old excipient paradigm outright because the same nominal excipient behaves differently depending on particle size, surface charge, PEG density, ionizable-lipid pKa, protein corona, cargo, and process. The EMA’s nanomedicine horizon work makes the essential point that standalone evaluation of nanocomponents can mislead because all components contribute to pharmacology and toxicity, and it highlights immune effects, hemocompatibility, biodistribution, thrombosis, complement activation, and CARPA-like reactions for intravenous nanomedicines (EMA, 2025). There is no harmonized guidance specific to novel lipid excipients, and nonclinical safety is often supported by studies of the full product or empty carriers. The need for human-relevant, animal-free methods tailored to such complex particles is one I flagged for nanomaterials early (Hartung, 2010; Hartung and Sabbioni, 2011). For nanomaterials in particular, physicochemical characterization is foundational rather than optional because the observed toxicity depends strongly on how the particle interacts with its surrounding medium. A central methodological requirement is therefore to characterize the particle in the biologically relevant matrix, including protein-corona formation, and to design assays and exposure models so that the *in vitro* medium interaction can be related to the human physiological environment rather than to an artificial dispersion.

The sensible NAM strategy tests four things in parallel rather than pretending that the excipient is separable: the component toxicology of each lipid, polymer, and surfactant; the empty carrier, to isolate carrier-driven effects; the final loaded product, to capture cargo-carrier interactions; the process and material attributes, including size distribution, surface properties, encapsulation, degradation, protein corona, and lot variability. The toolbox is human complement and cytokine assays, hemocompatibility, macrophage uptake, endothelial assays, hepatocyte and Kupffer co-cultures, proximal-tubule models, organ chips, and PBPK biodistribution. This is the route where the gap between the inactive-ingredient framework and biological reality is widest and where NAMs are most clearly the only sensible answer.

## 7 Vulnerable populations

The most compelling public-health argument for excipient NAMs is the protection of patients who cannot protect themselves. Children are not small adults, and IPEC notes plainly that excipients considered safe in adults can be problematic in children, with benzyl alcohol and ethanol as the textbook examples (IPEC, 2021; Clouser and Diseroad, 2022). The harms in Section 3 fall particularly hard on neonates and infants, while developmental exposures may also place the developing nervous system at risk (Smirnova et al., 2024).

Four opportunities follow directly. Age-specific PBPK turns an adult “safe history” into an actual pediatric margin of safety by representing the real differences in body water, renal clearance, hepatic metabolism, protein binding, and blood–brain barrier maturity rather than merely acknowledging them with an extrapolation factor. The reliability of these models depends on the quality of the underlying developmental-enzyme, renal-maturation, protein-binding, tissue-composition, and transporter data and on clinical calibration; model-derived margins therefore carry their own uncertainty. Just as a negative developmental-neurotoxicity finding should not be read as proof of safety, a favorable PBPK margin should be reported with its confidence bounds rather than as a single number. A NICU and pediatric cumulative-exposure model, built from the formulary and labels, identifies the avoidable fraction of the excipient burden documented in Section 4. Developmental neurotoxicity assessment can draw on the OECD *in vitro* battery^4^, with the critical caveat, which the OECD itself states, that negative findings must not be read as proof of absence of DNT potential; the battery informs weight of evidence and prioritization—it does not issue waivers (OECD, 2023; Smirnova et al., 2014). Moreover, ICH S11, which harmonizes nonclinical pediatric safety assessment, gives NAMs a clear role in deciding when juvenile animal studies actually add value and when age-specific exposure modeling plus targeted *in vitro* data suffice (ICH, 2020).

The posture I wish to stress is honest calibration. For pediatric excipient safety, the right use of a NAM is to prioritize, to derive a human-relevant point of departure, and to support read-across rather than to manufacture false reassurance. A negative DNT screen is a reason to deprioritize, not a clean bill of health. That discipline is what will earn these methods regulatory trust.

The same logic extends to geriatric patients, where reduced renal and hepatic function and heavy polypharmacy raise both accumulation risk and cumulative exposure, and to the critically ill, who receive prolonged infusions of PG-, ethanol-, and cyclodextrin-containing drugs. In each case the tool is the same: exposure modeling that represents the actual physiology of the actual patient.

## 8 Systemic organ toxicity: target uncertainty, do not oversell certainty

For systemic toxicity involving the liver, kidney, heart, immune, endocrine, and reproductive systems, the honest position is more measured than the enthusiasm sometimes heard at conferences. NAMs are not equally mature across these endpoints (Figure 3), and their best near-term role is to identify mechanisms, derive human-relevant points of departure, support read-across, and focus or reduce animal studies, not claim wholesale replacement for every chronic endpoint.

Where NAMs are strong, they are very useful. Hepatocyte spheroids, liver-on-chip systems, Kupffer-cell co-cultures, and bile-acid transport assays detect concentration-dependent liabilities of solubilizers, surfactants, polymers, and degradants. Renal proximal-tubule models address the cyclodextrin and accumulation questions. iPSC-derived cardiomyocytes, endothelial barrier assays, and platelet and coagulation assays serve intravenous excipients and nanocarriers. Steroidogenesis, receptor-transactivation, placental-transfer, and transcriptomic assays prioritize endocrine and reproductive concerns for chronic-use and pregnancy exposures. Microphysiological systems are now credible enough for these mechanistic purposes that integrated strategies combining organ chips, PBPK, and computational prediction can address questions traditionally handed to whole-animal studies (Marx et al., 2025; Hartung and Smirnova, 2025). A distinction should be kept explicit throughout: demonstrating concentration-dependent biological activity in a spheroid or organ chip is not the same as predicting clinically relevant toxicity. Relevance is established only when the active concentration is connected to free concentration, dosimetry, metabolism, and achievable human tissue exposure through *in vitro* to *in vivo* extrapolation and PBPK.

The highest-value systemic application is read-across within excipient families. PEGs, polysorbates, cyclodextrins, cellulose derivatives, methacrylate polymers, and lipid classes are families, not isolated molecules, and treating them as categories, with NAM data anchoring the grouping, can avoid repeating animal studies for every chain length, substitution pattern, or molecular-weight distribution. This is where mechanistic NAMs and read-across compound each other and where the inactive-ingredient framework now wastes the most resources.

## 9 From a test list to a safety passport: the integrated package

**FIGURE 4:**
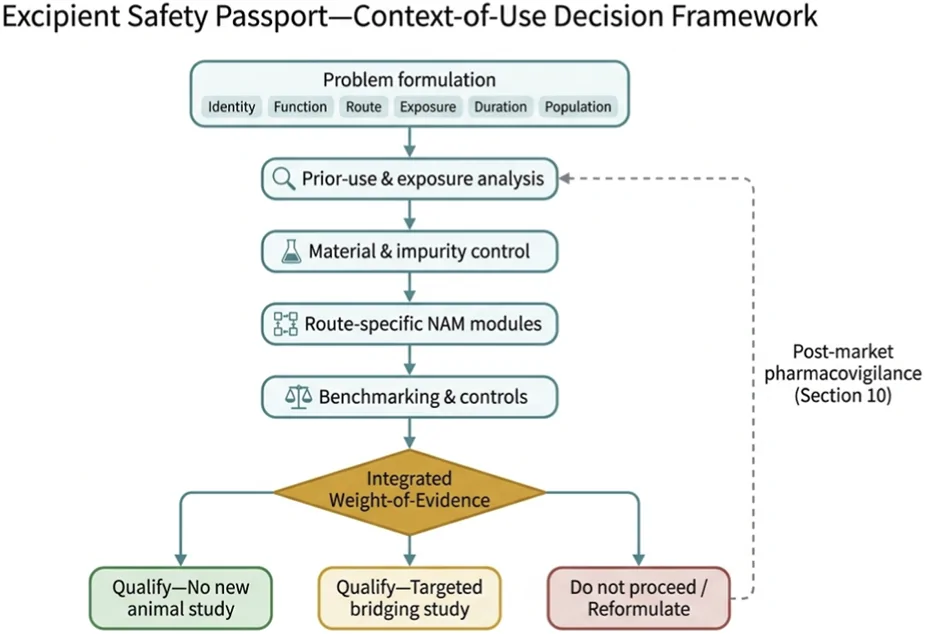
Excipient safety passport: a context-of-use decision framework. A vertical flow from problem formulation (identity, function, route, exposure, duration, and population) through prior-use and exposure analysis, material and impurity control, route-specific NAM modules, benchmarking and controls, to an integrated weight-of-evidence node, which reports both the direction of the decision and the associated confidence and residual uncertainty and which branches to one of three outcomes: qualify with no new animal study, additional targeted evidence needed (further NAM, analytical, exposure, or clinical data, and only a targeted bridging study where genuinely required), or “do not proceed/reformulate.” Residual-uncertainty planning, the seventh element of Section 9’s package, is shown as an explicit step that feeds these outcomes rather than being left implicit. A feedback arrow from post-market pharmacovigilance (Section 10) returns to the weight-of-evidence and hazard-characterization nodes to update exposure assumptions, confidence ratings, reference materials, and specifications. The figure renders the seven-part package of Section 9 as a single navigable diagram.

The pieces only become a system when they are organized around explicit decisions rather than around a menu of assays. As a persuasive NAM-based novel-excipient package, the content of the safety passport proposed in Section 2 has seven parts (Figure 4), and I lay them out because the structure is the contribution.

It begins with problem formulation: a precise statement of the excipient, its proposed function, route, exposure, duration, target population, product context, and why existing excipients are insufficient. The FDA’s novel-excipient pilot already asks for exactly these elements, which is convenient because they are also the inputs a NAM strategy needs (FDA, 2021). Second comes prior-use and exposure analysis, converting IID history, approved products, literature, and food or cosmetic experience into maximum daily exposure, local exposure, mg/kg exposure, and population-specific margins. Third is material and impurity control: full analytical characterization with ICH M7, Q3D, and Q3C applied and targeted testing only where alerts fire. Fourth are the route-specific NAM modules of Section 6, chosen because they answer a decision question and for no other reason. Fifth is the benchmarking and controls, using reference excipients with known clinical safety profiles, positive and negative controls, donor variability, concentration-response, and formulation-relevant conditions that the technical characterization the FDA draft explicitly demands, including attention to test-article interactions with assay materials (FDA, 2026).

Sixth, and central, is the integrated weight of evidence, which states plainly what the NAMs do: replace a traditional method, fill a gap, complement traditional data, justify not using a non-informative animal species, or support a borderline decision. The FDA framework recognizes all of these roles, and naming the role for each piece of evidence is what turns a pile of assays into an argument (FDA, 2025). Seventh is a residual-uncertainty plan that, where uncertainty genuinely remains, deploys targeted rather than default *in vivo* studies. The asymmetry here is the whole point: a higher-dose oral excipient supported by strong human exposure, negative impurity assessment, PBPK, and local gastrointestinal NAMs (Debad et al., 2024) may need no new animal study at all, while a chronic inhaled polymer with a genuine data gap may justify one short, well-targeted bridging study. The framework specifies animal studies where they actually reduce uncertainty and nowhere else. Three decision rules make the passport operable rather than aspirational. First, a defined minimum core dataset is required before any integrated conclusion is drawn. Second, discordant evidence is reconciled explicitly by weighting methods according to biological relevance and reliability and by defaulting to the more conservative interpretation when a genuine conflict cannot be resolved rather than being averaged away. Third, the output is not a binary certification but a graded conclusion that carries an explicit confidence level and a statement of residual uncertainty. Evidence tiers help here, distinguishing extensively characterized excipients with substantial human exposure from novel polymers, biological materials, or nanocarriers supported mainly by mechanistic and modeled evidence.

This is the same architecture I have advocated for evidence-based toxicology generally: define the question, assemble the most human-relevant evidence, weigh it transparently, and reserve new *in vivo* work for the residual uncertainty that nothing else resolves (Hoffmann and Hartung, 2006; Hartung, 2009; Hartung and Tsaioun, 2024). Excipients are simply an unusually clean place to apply it.

## 10 Make pharmacovigilance excipient-aware

A safety passport is a prospective instrument. It needs a retrospective complement because rare excipient reactions are precisely the events that prospective testing is worst at catching, and at present our surveillance systems are structurally blind to them. Adverse-event databases are organized around the active ingredient. When a patient reacts to “the drug,” the polysorbate, dye, or preservative that actually caused it is rarely recorded as the suspect, which is one reason why a substantial share of idiopathic anaphylaxis may be excipient-related and invisible (Malkawi and Altahrawi, 2025). A caveat must travel with any such system: detecting a statistical association between an excipient and an event is not the same as establishing causation (Hartung et al., 2026) because patients are exposed to many active and inactive ingredients at once. Attribution is strengthened by recurrence across unrelated products that share the excipient, formulation comparisons, de- and rechallenge, mechanistic plausibility, and lot-level and exposure information—exactly the fields an excipient-aware system should capture.

The fix is an information-architecture project, not a laboratory one. A modern system would make formulations searchable by excipient, route, amount, manufacturer, and lot, linking compendial identifiers (UNII and CAS), IID and label data, supplier, and lot metadata where available, electronic health record medication histories, allergy documentation, and spontaneous reports with natural-language processing of narratives that mention PEG, polysorbate, dyes, preservatives, sweeteners, or “inactive ingredients.” This would not replace preclinical testing; it would close the loop, feeding real-world signals back to refine PBPK assumptions, read-across categories, and the reference sets against which NAMs are calibrated. An excipient-aware exposome (Sillé et al., 2020; Hartung, 2023), in other words, is the surveillance counterpart of the safety passport, and the two should be built to talk to each other. Making excipient composition transparent and searchable also serves the public directly, and post-market vigilance will only generate strong signals if reporting is actively incentivized through clinician and patient reporting pathways and through manufacturer and formulary participation, making civil society a contributor to, and not merely a beneficiary of, excipient safety.

## 11 Implementation and the risk of getting it wrong

A vision without a sequence is a wish, so I close with a phased agenda and an honest account of the failure modes.

In the near term, indicatively the first 1 to 2 years, the actionable wins are unglamorous and real: standardize excipient maximum-daily-exposure and PBPK templates; apply ICH M7, Q3D, and Q3C systematically to excipient impurities and degradants; use the already-accepted skin, eye, sensitization, and phototoxicity NAMs for topical, ocular, and dermal excipients; replace rabbit pyrogen testing with risk-based *in vitro* pyrogenicity wherever pharmacopoeially permitted; build parenteral panels for the monocyte activation test, complement, cytokines, and hemocompatibility. Most of this requires adoption, not invention. In the following phase, indicatively years 3 to 5, the work is platform qualification: route-specific batteries for inhalation, nasal, ocular, and injectable excipients; excipient reference sets with known clinical outcomes; read-across categories for polymer families, surfactants, PEGylated materials, cyclodextrins, and lipids; pediatric cumulative-exposure tools linked to formularies; parallel-testing nanocarrier platforms. Beyond roughly 5 years lies the structural transformation: portable excipient safety passports maintained by manufacturers and referenced across sponsors, confidential but regulator-accessible data-sharing to end duplicative studies, harmonization across FDA, EMA, ICH, OECD, IPEC, and the pharmacopeias, and post-market signal detection that continuously updates safe limits and NAM reference sets.

The cautions are as important as the agenda because the failure modes are specific. The first is false reassurance, the cardinal scientific danger. A negative NAM result is only meaningful within its context of use, and for excipients the confounders are unusually severe: poor solubility, adsorption of surfactants and lipids to plastic and to organ-chip materials, protein binding, missing metabolism, lack of chronic exposure, and direct interference of the test article with the assay readout. The FDA draft rightly calls out test-article and platform interactions, and these are most acute for exactly the surfactants, polymers, lipids, and nanoparticles that excipients comprise (FDA, 2026). A clean result from a compromised assay is worse than no result. The second danger is regulatory fragmentation: as long as five agencies and three pharmacopeias hold subtly different expectations, sponsors will default to the most conservative, animal-heavy path, and the EMA’s own horizon work notes that NAM use in submissions remains limited partly because developers are cautious and regulators have limited experience with cutting-edge data (EMA, 2025). The third danger is data access: while excipient safety and composition sit locked in supplier files and Drug Master Files, sponsors will keep repeating studies they could inherit. A trusted excipient data commons, with confidential regulatory access and public summaries, would be one of the highest-impact infrastructure investments in the entire field, and it is an organizational, not a scientific, decision. It helps to locate these failure modes where they actually arise, in material characterization, assay performance, biological relevance, exposure extrapolation, evidence integration, and post-market interpretation, so that each safeguard maps onto a specific stage of the passport. Constructing the reference sets that anchor these judgments is itself a nontrivial scientific task because known safe and known harmful are context-dependent classifications; reference excipients must therefore be selected with explicit, stated clinical-outcome criteria rather than assumed. A further danger is data quality at the source: computational and read-across models are only as trustworthy as the data feeding them, and the documented rise of fabricated, manipulated, or AI-generated data in the scientific record (Sanderson, 2024; Sharifi, 2025; Naddaf, 2025; Omar et al., 2025; Jian and Yaeger, 2025) makes the provenance, curation, and quality control of training and reference data themselves safety-critical.

## 12 Conclusion

The word “inactive” has outlived its usefulness and, in too many cases, its victims. Excipients are not inert, and the past decade has cost real lives demonstrating it, from PEG anaphylaxis to neonatal gasping syndrome to the recurrence, 85 years after sulfanilamide, of children dying from diethylene glycol. The conventional response, a 2005 guidance and product-by-product animal testing, has neither prevented these harms nor matched the science to the question.

My argument has been that excipients are not a hard case for NAMs but a favorable one because excipient risk is contextual rather than intrinsic, and context, route, dose, duration, formulation, impurity, and patients are exactly what exposure science, computational chemistry, and human *in vitro* systems were built to address. The first wave is available now and, allowing for the differing scope and readiness of its components, is largely ready for adoption: impurity qualification by ICH M7 and Q3D and Q3C, maximum-daily-exposure and PBPK modeling, the accepted local-tolerance NAMs, *in vitro* pyrogenicity, and parenteral immune and blood-compatibility panels. The second wave—inhalation, pediatrics, nanocarriers, and chronic organ toxicity through fit-for-purpose batteries—is where qualification effort should now concentrate. The organizing structure for all of it is the excipient safety passport: a context-of-use, exposure-led, mechanistic document that travels across products, routes, and sponsors, updated by NAM data, clinical evidence, and real-world surveillance. Crucially, the passport is modular and adapted to material class, route, exposure, formulation, and population; it is meant to replace a universal testing checklist, not become one.

The endpoint is a discipline in which excipient safety is no longer a static checklist inherited from a database but is a human-relevant, quantitative argument that we can actually defend. We have called these ingredients inactive for a century. The honest course now is to find out, with the best human-relevant tools we have, what they actually do.

## Footnotes

1
https://www.fda.gov/drugs/drug-approvals-and-databases/inactive-ingredients-database-download

2
https://www.fda.gov/science-research/science-and-research-special-topics/new-approach-methodologies-nams

3
https://www.fda.gov/regulatory-information/search-fda-guidance-documents/general-considerations-use-new-approach-methodologies-drug-development

4
https://www.oecd.org/en/topics/sub-issues/testing-of-chemicals/in-vitro-assays-for-developmental-neurotoxicity.html
